# Thyroid Dysfunction in Chinese Patients with Chronic Hepatitis C Treated with Interferon Alpha: Incidence, Long-Term Outcome and Predictive Factors

**DOI:** 10.5812/hepatmon.6390

**Published:** 2012-09-30

**Authors:** Zehui Yan, Ke Fan, Yi Fan, Xiaohong Wang, Qing Mao, Guohong Deng, Yuming Wang

**Affiliations:** 1 Institute of Infectious Diseases, Southwest Hospital, the Third Military Medical University, Chongqing, China

**Keywords:** Hepatitis C, Interferon-Alpha

## Abstract

**Background:**

Thyroid dysfunction (TD) represents an extra-hepatic manifestation of chronic hepatitis C (CHC) and it may also be a side effect of interferon-alpha (IFN-α) based treatment. However, previous studies have shown a wide variation in the incidence of TD in patients with CHC. Furthermore, the long-term outcomes and the predictive factors of TD in patients who receive IFN-α based treatment have still not been fully studied.

**Objectives:**

The purpose of this study was to describe the incidence and long-term outcomes of TD in Chinese patients with CHC receiving IFN-αbased treatment. We also aimed to identify the predictive factors of TD associated with this type of therapy.

**Patients and Methods:**

A retrospective case-series study of 592 consecutive CHC patients with normal baseline thyroid functions, who received IFN-αbased therapy, was performed. Thyroid function was assessed at baseline and every three months during treatment, as well as in the follow-up after cessation of therapy. The incidence and long-term outcomes of TD were observed. The prevalence of pretreatment thyroid peroxidase antibodies (TPOAb) were assayed in a sex- and age-matched nested case-control study. Multivariable stepwise regression analysis was used to explore the independent effects of the baseline factors, on the incidence of TD.

**Results:**

At the end of the IFN-αbased therapy, 68 patients (11.5%) in the study had developed TD, 58 patients (85.3%) presented with subclinical TD, and only 10 patients (14.7%) developed overt thyroiditis. The thyroid function of 46 patients (67.8%) spontaneously returned to normal in the six months of follow-up and only three patients (4.4%) had persistent overt TD symptoms after the 24 month follow-up period. Multivariate stepwise analysis suggested that gender and pretreatment TPOAb were the independent factors related to the incidence of TD. Both female patients (OR, 4.31; 95%CI, 2.06–7.31; P = 1.26×10-4) and participants with a positive pretreatment TPOAb (OR = 3.9, 95%CI, 1.72–8.54, P = 0.008) had an increased risk for the development of TD.

**Conclusions:**

The incidence of TD in Chinese patients with CHC during IFN-αbased therapy was 11.5%, the majority of which was subclinical, while only a very small group had long-term overt TD requiring ongoing medical therapy. Female gender and pretreatment TPOAb positivity are risk factors for the development of TD during IFN-αbased therapy.

## Background

Globally, the hepatitis C virus (HCV) has infected an estimated 170 million people, most of whom are chronically infected with a high risk of cirrhosis and hepatocellular carcinoma and they serve as a reservoir for transmission to others (1, 2). Interferon-alpha (IFN-α) based antivirus therapy, regular IFN-α or pegylated interferon alfa (Peg-IFN-α), in combination with ribavirin or alone, is recognized as being a highly effective treatment for patients with chronic hepatitis C (CHC).This therapy results in a sustained virologic response (SVR) in 40–50% of patients with genotype 1, and around 80% in those infected with genotype 2 and 3 (3, 4). Despite their favorable efficacy, these IFN-αbased regimens are also accompanied by many well-known adverse effects, including; fever, depression, anemia, neutropenia, thrombocytopenia and endocrine side effects (5-7). The most commonly documented endocrine side effect of these IFN-αbased regimens, outside of the liver, is the production of autoantibodies and the development of thyroid dysfunction (TD) (8-10). The mechanisms involved in TD still remain unclear. It has been argued that virus-factors especially HCV itself, may predispose patients to the development of TD, although it must be noted that some patients with CHC may have experienced thyroid problems before treatment. TD is less likely to develop in patients with a chronic hepatitis B infection, who are treated with interferon alpha than in a CHC virus infection, despite the use of higher doses of interferon alpha for the treatment of hepatitis B virus (HBV) (11). In addition, IFN-α does not usually predispose patients to the development of TD during or after treatment. In that case, a HCV infection and IFN-α may play a synergistic role in inducing thyroid disease during antiviral therapy. Research suggests that IFN-α induces an autoimmune reaction that leads to the development of anti-thyroid antibodies and subsequent TD (10). Furthermore, IFN-α has direct inhibitory effects on thyroid hormone synthesis, release and metabolism, as well as abnormal expression of major histocompatibility antigens on thyroid cells (12). Apart from IFN-α, there are also data suggestive of immune modulatory effects of ribavirin on the thyroid gland (13, 14). In addition, a distinct effect of IFN-α therapy on intra thyroidal organification of iodine is suggested to cause TD (10, 15). Previous studies have shown a wide variation of TD incidence in patients with CHC during and after IFN-αbased therapy, at a rate of 2.5% to 35% in different countries (16-28). This variability can be attributed either to an underestimation of the true prevalence of TD or to the diverse genetic predisposition of the subjects (29). Differing definitions of TD used in different studies may also play a role in the TD described. The spectrum of TD involves hypothyroidism and hyperthyroidism. It can also be subclinical or overt. However, the long-term outcomes of TD after IFN-αbased therapy remain controversial. Some reports have shown reversible TD in all patients (30, 31), while others show only partial reversibility by the end of follow-up (11, 20, 32-35). Previous studies have also shown that IFN-α related TD is associated with female gender, the presence of thyroid autoantibodies, and an oriental background (11, 36, 37). It has been shown that the emergence of thyroid autoantibodies, such as thyroid peroxidase antibody (TPOAb), may predict a possible underlying autoimmune process and they are useful indicators of present or future TD. However, there have only been a few reports addressing the predictive role of autoantibodies on the occurrence of TD in CHC patients receiving IFN-αbased therapy (20, 26, 33, 38, 39).

## Objectives

Reports about the incidence, long-term outcomes and predictive factors of TD in Chinese populations with CHC treated with IFN-α are scarce, except for two studies from Taiwan (20, 22). The purpose of this study was to describe the incidence and long-term outcomes of TD in Chinese patients with CHC receiving IFN-αbased treatment. We also aimed to identify the predictive factors of TD associated with IFN-αbased therapy on CHC. For this purpose we conducted a prospective cohort study in a single center. We also conducted a nested case-control study in order to estimate the role of TPOAb in predicting the risk of TD development.

## Patients and Methods

### 3.1. Study Participants

A total of 674 individuals with a HCV infection who were registered and followed-up at the Department of Infectious Diseases of Southwest Hospital (the largest hepatology center in southwest China, which treats approximately 2500 inpatients and 75 000 outpatients per year) from January 2004 and June 2011 were included in the primary selection. All subjects provided their informed consent to participate in the study, as approved by the Ethical Committee of the Southwest Hospital, Chongqing, China. Patients with the following conditions were excluded; (i) coinfection with human immunodeficiency virus (HIV) or HBV, (ii) peripheral blood leukocyte count < 3×109/L or platelet count < 70×109/L or hemoglobin level lower than 100 g/L, (iii) pregnancy and lactation, (iv) concomitant serious medical illnesses, such as; malignancy, severe cardiopulmonary disease, or uncontrolled diabetes mellitus. The diagnosis of CHC was made by a persistent or intermittent elevation of alanine aminotransferase (ALT, the upper limit of normal ALT is 40 IU/L) over a six-month period, anti-HCV positivity and detection of HCV-RNA in the sera. Finally, a total of 592 consecutive patients (the participation rate was 87.8%) with CHC and normal baseline thyroid functions were enrolledin this prospective cohort study. The baseline characteristics, routine laboratory data and virological information of the 592 patients were collected from clinical records and short telephone interviews when necessary. Among them, 323 patients (54.6%) were male and 269 patients (45.4%) were female, their ages ranged from 19 to 58 years with a mean age (± SD) of 39.2 (± 10.3) years. TSH, FT4 and FT3 levels before antiviral treatment were known for all of the study participants and these were within normal ranges.

### 3.2. IFN-αBased Therapy and Assessment of Response to Therapy

Three kinds of IFN-αbased therapy regimen; regular IFN-α monotherapy, regular IFN-α plus ribavirin, and Peg-IFN-α combined with ribavirin, were received by patients for a period of 24 to 48 weeks according to their HCV genotype(48 weeks for patients infected with HCV genotype 1, and 24 weeks for patients infected with HCV genotype 2, 3 and 6). The dosage of regular IFN-α ranged from 3 to 5 MU twice weekly. The dose of Peg-IFN-α-2a was 180μg or 135μg once a week, and that of Peg-IFN-α-2b was 50μg or 100μg, also administered once a week. Eighty-eight of the 592 patients received IFN-α monotherapy, while ribavirin was co-administered in 504 patients at a daily dose of 600 to 1200 mg, according to their bodyweight. Of the 504 patients, 161 patients received regular IFN-α plus ribavirin therapy, while 343 patients received the combination therapy with Peg-IFN-α plus ribavirin. The duration of follow up was calculated as the time from the initiation of therapy until the last time that the patient was seen at the outpatient clinic. Dose adjustments or therapy interruptions were made according to the specific characteristics of each individual patient and on specialists’ recommendations. In cases of permanent thyrotoxicosis (in particular Graves’ disease), IFN-αbased therapy was discontinued.

### 3.3 Laboratory Tests and Thyroid Function Assessments

All patients were evaluated; clinically, hematologically, biochemically and serologically at baseline. Routine biochemical and hematological tests were performed using automated techniques. HCV antibodies were detected using third generation commercial enzyme linked immune sorbent assay kits, INNO-LIA HCVAb III update Immuno Assay (LIA, Innogenetics s.r.l., Gent, Belgium). The HCV RNA load was tested on the seroreactive samples using quantitative real-time polymerase chain reaction (PCR) (ABI Prism 7000, Applied Biosystem, Foster City, CA). HCV genotype was determined as described in our previous research, by direct sequencing of the nested PCR with two sets of conserved primers deduced from the core-envelope1 (CE1) region of the HCV genome (40). Thyroid function tests, including serum thyrotropin (TSH), total thyroxine (TT4) and free thyroxine (FT4), total triiodothyronine (TT3) and free triiodothyronine (FT3) were performed by an ultrasensitive immune chemiluminescent non-competitive assay (ICMA) (Immnulite 2500 DPC, USA). TD was defined as any value of these markers (TSH, TT3, FT3, TT4 and FT4) which was greateror less than the normal values. The four types of TD recognized in our study were defined as follows; (i) subclinical hyperthyroidism, TSH < 0.3mIU/L accompanied with normal FT4 and FT3; (ii) overt hyperthyroidism, TSH < 0.3mIU/L accompanied with increased FT4 or FT3; (iii) subclinical hypothyroidism, TSH > 5.0mIU/L accompanied with normal FT4 and FT3; (iv) overt hypothyroidism, TSH > 5.0mIU/L accompanied with decreased FT4. Thyroiditis was defined as hyperthyroidism or subclinical hyperthyroidism diagnosed initially, which subsequently converted to hypothyroidism or subclinical hypothyroidism during follow-up. All patients with TD were followed-up by means of TSH (FT4 and FT3 when available) along with clinical assessment for a period of three months to six years after discontinuation of the therapy. Patients received drug replacement therapy for TD when TSH > 10mIU/L.

Since the TPO antibody status of the patients were not prospectively tested at baseline in our study, we performed laboratory tests of the pretreatment TPOAb using stored frozen sera obtained before the disease developed in our nest-case control study. A conventional case-control approach was used to select sex- and age-matched cases and controls in the retrospective cohort. The nest-case control study was economical and produced data capable of indicating a good causal relationship. TPOAb was measured using these stored sera by a micro-enzyme immunoassay method (Roch, USA) and an elevated TPOAb up to 60 IU/L was defined as being TPOAb positive.

### 3.4 Statistical Analysis

Statistical analysis was performed using SPSS software (version 9.0; SPSS Inc, Chicago, IL). Quantitative variables were expressed as mean ± standard deviation when the data was normally distributed, while variables were expressed as a median (range) when the data was not in a normal distribution. Quantitative variables were compared by a Student’s test. Frequencies were calculated for categorical variables. The χ2 test was used, with Yates’ correction where applicable, to compare categorical variables. Univariable logistic regression analysis and multivariable logistic stepwise regression analysis were used to explore the independent effect of the baseline factors on the incidence of TD, with the difference between groups reported with 95% confidence intervals (CIs). A 2-sided P value less than 0.05 was considered to be significant.

## Results

### 4.1. Incidence and Long-Term Outcome of Thyroid Dysfunction

At the end of IFN-αbased therapy, 68 patients (11.5%) out of the total study group developed biochemical TD (TSH < 0.3 or > 5.0 mIU/L), 47 women and 21 men were affected. The time in which themajority of TD onset cases occurred in our study was before the 6th month after initiation of IFN-αbased HCV treatment. Of the 68 cases with TD, 39 patients (57.4%) developed TD in the first three months, 21 patients (30.9%) in the following three months, while only eight patients (11.8%) developed TD in the final six months after initiation of IFN-αbased HCV treatment. The cumulative incidence at three, six and 12 months after initiation of IFN-αbased HCV treatment was 6.6%, 10.1% and 11.5%, respectively. Of the 68 cases with TD, thirty patients (44.1%) developed an initially suppressed serum TSH concentration (< 0.3 mIU/L), while 38 patients (55.9%) developed an elevated serum TSH (> 5.0 mIU/L). Transient and subclinical TD accounted for 85.3% of all TD observed and only 10 patients (14.7%) presented with overt thyroiditis. Among the 30 patients with hyperthyroidism, 26 patients (86.7%) had transient thyroiditis and presented with subclinical hyperthyroidism, while only four patients presented with overt hyperthyroidism. A total of 38 patients with hypothyroidism, and 32 hypothyroid subjects (84.2%) experienced a transient episode and subsequently presented with subclinical hypothyroidism, while only six patients developed overt hypothyroidism, requiring levothyroxine replacement therapy. All of the 68 patients with biochemical TD were followed-up after cessation of IFN-αbased therapy. The mean follow-up period was 36 months (ranging from 6 to 96 months). The thyroid function of 46 patients (67.8%) became normal spontaneously in the six month follow-up period after the cessation of IFN-αbased therapy. Among the 22 persistent TD patients (32.4%), except for one patient who had not finished the 12 month follow-up, five patients became normal spontaneously and two cases became normal by receiving antithyroid drugs in the 12 month follow-up. Among the remaining 14 patients with TD, three patients had not finished the 24 month follow-up and 11 were followed up at least 24 months later. At the 24 month follow-up after cessation of combination therapy, three patients had recovered from their biochemical thyroid function by receiving antithyroid drugs, while persistent biochemical TD was found in only eight patients. In these eight patients, five patients had asymptomatic TD, including subclinical hyperthyroidism in two patients and subclinical hypothyroidism in three patients. Of the remaining three patients, two patients who had been diagnosed with Graves’ disease had symptomatic hyperthyroidism and received antithyroid drugs and one patient was diagnosed with Hashimoto thyroiditis which manifested as symptomatic and theyreceived a thyroxine supplement.

### 4.2. Prediction of Thyroid Dysfunction

The mean age of patients developing TD (41.4 ± 9.5 y) and those remaining euthyroid (38.9 ± 11.4 y) during treatment in the total population were similar (P = 0.823). There were no significant differences between patients with TD and those without TD in respect to their; ALT levels, HCV RNA levels, cirrhosis status and TSH levels (all P > 0.05). Between patients with and without TD, statistically significant differences were observed with respect to gender. The incidence of TD in women (47 of 269, 17.5%) was higher than that in men (21 of 323, 6.5%; P = 3.07×10-5). The patients known to have HCV genotypes 1, 2, 3 and 6 had similar incidences of developing TD (12.3%, 9.7%, 11.8% and 9.4%, respectively, P = 0.856). No significant difference was found for each of the HCV genotypes (all P > 0.05). The incidence rate of TD in patients infected with genotype 1 (12.3%) seemed higher than that found in patients infected with genotypes 2, 3 and 6 (10.7%), but the difference was not statistically significant (P = 0.546). Seven of the 88 patients (8.0%) who had received regular IFN-α monotherapy developed TD, while 14 out of 163 patients (8.6%) who received regular IFN-α plus ribavirin combination therapy and 47 of 341 patients (13.8%) who received Peg-IFN-α plus ribavirin combination therapy also developed the condition. There were no significant differences for the incidence rates of TD among the three IFN-αbased therapy regimens (P = 0.145). However, a borderline significant difference (P = 0.047) was observed when comparing the incidence rate of TD between the patients who received regular IFN-α-based therapy (8.4%) and the patients receiving Peg-IFN-αbased therapy (13.7%). In a univariable logistic regression analysis of factors associated with TD during IFN-αbased therapy; gender, age, HCV genotype, cirrhosis, and regimen of IFN-α-based therapy were analyzed; only gender was significantly associated enough to justify an inclusion in the equation(OR, 3.64; 95%CI, 1.7–5.8, P = 0.0001). We also conducted multivariate stepwise analysis, included in the model were the following preselected variables; gender (male vs. female), age (aged < 40 years vs. ≥ 40 years), HCV genotype (genotype 1 vs. 2/3 and 6), absence of cirrhosis (with vs. without) and IFN-αbased therapy (regular IFN-αbased therapy vs. Peg-IFN-αbased therapy). According to multivariate stepwise analysis, again, gender was the only independent factor that related to the incidence of TD and female gender was also significantly associated with the incidence of TD (OR, 4.31; 95% CI, 2.06–7.31; P = 1.26×10-4).

### 4.3. Sex-and Age-Matched Nested Case-Control Study

To assess the implications of thyroid autoantibodies in the prediction of TD, we performed a sex-and age-matched nested case-control study and assayed serum thyroid autoantibodies before treatment. The results of our nested case-control study are shown in Table 1 (47 women and 21 men in both groups). The mean age of the case (41.4 ± 9.5 years) and control (42.1 ± 10.4 years) groups was comparable (P = 0.973). Overall, the prevalence of pretreatment TPOAb was 14.0% (19 of 136). In univariate analysis, there were significantly higher (nearly fourfold) positive rates of pretreatment TPOAb in patients with TD (22.1%, 15 of 68) when compared with patients without TD (5.9%, 4 of 68) (P = 0.007). We also performed a multiple logistic regression analysis that included; age, HCV-RNA levels, HCV genotype, cirrhosis, regimens of IFN-αbased therapy, and pretreatment TPOAb, we found that pretreatment TPOAb was the only independent factor that was associated with the development of TD (OR = 3.9, 95% CI = 1.72–8.54, P = 0.008 ).

**Table 1:** Sex and Age-Matched Nested Case-Control Study of Thyroid Dysfunction in Chronic Hepatitis C Patients Received IFN-α-Based Therapy

	Patients With TD (n = 68)	Nested Control Group (n = 68)	P value
**ALT[Table-fn fn306] level , IU / L, median (range)**	152 (69-267)	171 (56-301)	0.238 a
**HCV [Table-fn fn306] RNA , IU/mL ×103, median (range) **	625 (0.20-14200)	607 (0.30-1250)	0.553 a
**Diagnosis, No. (%) **			0.598 b
**CHC without cirrhosis **	43 (63.2)	40 (58.8)	
**LC **	25 (36.8)	28 (41.2)	
**HCV genotype, No. (%) **			0.605 b
**Type 1b **	36 (52.9)	39 (57.4)	
**Non-type 1 (Type 2/3/6) **	32 (47.1)	29 (42.6)	
**IFN-α- based therapy type, No. (%) **			0.463 b
**Regular IFN-α monotherapy**	7 (10.3)	5 (7.4)	
**Regular IFN-α + ribavirin **	14 (20.5)	13 (19.1)	
**Peg-IFN-α + ribavirin **	47 (69.1)	50 (73.5)	
**Pretreatment TPOAb, No. (%) **			0.007 b
**Positive**	15 (22.1)	4 (5.9)	
**Negative**	53 (77.9)	64 (94.1)	

Abbreviations: ALT, alanine aminotransferase; CHC, chronic hepatitis C;HCV, hepatitis C virus; IFN-α, interferonalpha; LC, liver cirrhosis;Peg-IFN-α, pegylated interferonalpha;TD, thyroid dysfunction;TPOAb, thyroid peroxidase antibody.

^a^P value was given for the comparison between patients without TD and patients with TD by a Student’s t-test.

^b^P value was given for the comparison between patients without TD and patients with TD by a χ2 test.

## Discussion

Previous studies have shown that the incidence of TD associated with IFN-αbased therapy differs markedly between countries (16-28, 32, 35, 39, 41-46), with the lowest incidence described in Brazil and the highest was found in Poland in studies of adult subjects (43, 45). In the present study of a large Chinese cohort, the incidence of TD during IFN-αbased therapy for patients with CHC was 11.5%. The incidence of TD in our study is higher than the mean incidence (approximately 6%) reported in a meta-analysis study (10), but similar to the incidence of the study from Taiwan (22). The percentage of hypothyroidism (55.9%) was higher than that of hyperthyroidism (44.1%) as well as in most previous studies (22, 26, 34, 47). However, a study from southern Taiwan also found that the predominant type of TD during IFN-αbased therapy was hyperthyroidism (48). In our study, over half of the patients with TD (57.4%) developed TD in the first three months and the majority (88.2%) developed TD in the first six months after initiation of IFN-αbased HCV treatment. This result is different to data from a Greek study (26). We think there are some possible reasons for the variability of TD incidence and manifestation among different populations. First, the diverse genetic predisposition of the subjects may be one of the dominant factors attributed to the variability of TD incidence. For example, recent studies from Australia and the United Kingdom demonstrate that Asian ethnicity is an independent risk factor for the development of TD with a higher incidence than other ethnicities while on IFN-αbased therapies (17, 21). Second, iodine status in different cohorts studied may also play a role in the variation in TD described. The different manifestation of TD can be further explained by variations in dietary iodine intake in the populations studied. In general, high iodine intake is associated with hypothyroidism, whereas low iodine intake is related to hyperthyroidism (49). Third, differing definitions of TD used in different cohort studies may overrate or underestimate the true prevalence of TD. The outcome of patients with TD in previous studies and data are controversial. In some reports, they did not use a long enough follow-up period to fully evaluate thyroid function after treatment, some showed reversible TD in all patients (30, 31), while others showed only partial reversibility by the end of follow up (11, 20, 32-35). Even in the studies with a long-term follow-up to 24 months, the long-term outcomes of patients with TD remain controversial. Long-term follow-up in two studies demonstrated that a small group of patients had developed chronic thyroiditis and subclinical hypothyroidism, but no patients displayed overt TD by the end of follow-up (36, 38). However, a recent study with long-term follow-up showed that more than half of the patients with IFN-α-induced TD develop permanent thyroid disease(26). We also evaluated the thyroid function of patients with TD in a long follow-up period after discontinuation of antiviral treatment. In the present study, thyroid function of more than half of the patients (67.8%) became normal spontaneously in the six month follow-up after cessation of IFN-αbased therapy and only eight patients (11.8%) were affected by biochemical TD at the 24 month follow-up after cessation of therapy.

The causes for the different outcomes in various studies are still unknown, ethnic, environmental and genetic predisposition may also have played a role. In our study, age and HCV genotype were demonstrated to be of little use in the prediction of TD incidence, which is similar to most previously studies(17, 21, 22, 33), but there is no consensus in some of the other studies(25, 48). Panva et al. observed a higher prevalence of HCV genotype 1 in patients who developed TD (25). In the study by Hsieh et al., mixed genotypes of CHC were related to IFN-α-induced TD in univariate analysis, but these were not significant in multivariate analysis (48). They suggested that a portion of the HCV genome could share a partial sequence homology in a few amino acid segments with thyroglobulin and microsome, rendering HCV patients susceptible to autoimmune thyroid diseases. This hypothesis could explain the relationship between viral genotype and the predisposition to developing thyroid diseases in patients infected by virus C genotype 1, but this was not verified in our patient population. However, we should note that the number of patients enrolled in a study is crucial in order to investigate significant correlations. Theoretically, the HCV genotypes with lower frequencies in the cohort may be under-represented in a small sample and the true incidence of TD in patients with these genotypes may be higher than observed. Thus, another possible reason for the differences in the relationship with the HCV genotype, may be the relative number of subjects representing each of the various HCV genotypes in different studies, as the HCV genotype has also been shown to have unique patterns of geographic distribution(40).

To date, only a few studies have evaluated the incidence of TD in HCV infected patients treated with different IFN-αbased therapy regimens. Previous research has found that the mean incidence of TD in patients treated with interferon alpha and ribavirin combination therapy (12.1%) is higher than in those treated with interferon alone (6.6%) (50). In the present study, although a borderline significant result (P = 0.048) was observed, when comparing the incidence rate of TD between the patients who received regular IFN-αbased therapy (8.4%) and the patients who received Peg-IFN-αbased therapy (13.7%), there was no significant difference in the incidence of TD among those treated with the three different IFN-αbased therapy regimens. Furthermore, in logistic regression analysis or multivariate stepwise analysis, the type of IFN-αbased therapy regimens was not the independent factor associated with the development of TD. Our results are identical to the study from Taiwan (22), which suggests that ethnic and genetic predisposition may play an important role in the incidence of TD.

In the present study, gender was an independent factor in predicting the occurrence of TD, and females had an increased risk for the development of TD during IFN-αbased therapy. Many studies have reached similar conclusions(11, 17, 21, 22, 33, 48), although some studies did not find this correlation of gender with TD (51-53). Since immune reactivity is greater in females than in males and sex hormones influence the onset and severity of immune-mediated pathological conditions, we think that the hormonal status of females might be one of the possible causes that females appear to be more susceptible to IFN-αinduced TD than males. Earlier evidence has suggested that the sex hormones not only act via the thymus gland, but also influence the immune system by acting on several non-classic target sites including the thyroid (54). We found that the pretreatment TPOAb was the independent factor associated with the development of TD and patients testing positive for pretreatment TPOAb had a 3.9 fold higher risk of developing TD. This result is in agreement with previous reports (17, 20, 26, 38, 51, 55). TPO antibody status findings support a strong foundation for an immunological basis for the development of TD during IFN-αbased therapy. The hepatitis C virus itself has been hypothesized to have direct effects on thyrocytes and induce thyroid autoantibody production (56). Furthermore, IFN-α activates lymphocytes leading to increased cytokine production, and the induction of thyroid autoantibodies (29). In conclusion, our study has found that the incidence of TD in a Chinese cohort of patients with CHC during IFN-αbased therapy was 11.5%, the majority of which was subclinical. Long-term thyroid function of the majority of patients may become normalized spontaneously after cessation of IFN-αbased therapy, while only a small group of patients (1.4%) had persistent long-term overt TD that required ongoing medical therapy. Female gender and pretreatment TPOAb positivity were risk factors for the development of TD during IFN-αbased therapy in the present study. However, as a study from a single center, a bias may exist in our results. Our findings need to be confirmed by data from a much larger, multicenter and long-term follow-up prospective studies.

## References

[1] Negro F, Alberti A (2011). The global health burden of hepatitis C virus infection. Liver Int.

[2] Shepard CW, Finelli L, Alter MJ (2005). Global epidemiology of hepatitis C virus infection.. Lancet Infect Dis.

[3] Ghany MG, Strader DB, Thomas DL, Seeff LB (2009). Diagnosis, management, and treatment of hepatitis C: an update.. Hepatology.

[4] McHutchison JG, Lawitz EJ, Shiffman ML, Muir AJ, Galler GW, Mc-Cone J (2009). Peginterferon alfa-2b or alfa-2a with ribavirin for treatment of hepatitis C infection.. N Engl J Med.

[5] Fried MW, Shiffman ML, Reddy KR, Smith C, Marinos G, Goncales FL, Jr. (2002). Peginterferon alfa-2a plus ribavirin for chronic hepatitis C virus infection.. N Engl J Med.

[6] Manns MP, McHutchison JG, Gordon SC, Rustgi VK, Shiffman M, Reindollar R (2001). Peginterferon alfa-2b plus ribavirin compared with interferon alfa-2b plus ribavirin for initial treatment of chronic hepatitis C: a randomised trial. Lancet.

[7] Sokal EM, Bourgois A, Stephenne X, Silveira T, Porta G, Gardovska D (2010). Peginterferon alfa-2a plus ribavirin for chronic hepatitis C virus infection in children and adolescents.. J Hepatol.

[8] Antonelli A, Ferri C, Ferrari SM, Colaci M, Sansonno D, Fallahi P (2009). Endocrine manifestations of hepatitis C virus infection.. Nat Clin Pract Endocrinol Metab.

[9] Antonelli A, Ferri C, Pampana A, Fallahi P, Nesti C, Pasquini M (2004). Thyroid disorders in chronic hepatitis C.. Am J Med.

[10] Prummel MF, Laurberg P (2003). Interferon-alpha and autoimmune thyroid disease.. Thyroid.

[11] Fernandez-Soto L, Gonzalez A, Escobar-Jimenez F, Vazquez R, Ocete E, Olea N (1998). Increased risk of autoimmune thyroid disease in hepatitis C vs hepatitis B before, during, and after discontinuing interferon therapy.. Arch Intern Med.

[12] Roti E, Minelli R, Giuberti T, Marchelli S, Schianchi C, Gardini E (1996). Multiple changes in thyroid function in patients with chronic active HCV hepatitis treated with recombinant interferon-alpha.. Am J Med.

[13] Dixit NM, Perelson AS (2006). The metabolism, pharmacokinetics and mechanisms of antiviral activity of ribavirin against hepatitis C virus.. Cell Mol Life Sci.

[14] Snell NJ (2001). Ribavirin--current status of a broad spectrum antiviral agent.. Expert Opin Pharmacother.

[15] Tomer Y, Blackard JT, Akeno N (2007). Interferon alpha treatment and thyroid dysfunction.. Endocrinol Metab Clin North Am.

[16] Andrade LJ, Atta AM, Atta ML, Mangabeira CN, Parana R (2011). Thyroid disorders in patients with chronic hepatitis C using interferonalpha and ribavirin therapy.. Braz J Infect Dis.

[17] Costelloe SJ, Wassef N, Schulz J, Vaghijiani T, Morris C, Whiting S (2010). Thyroid dysfunction in a UK hepatitis C population treated with interferon-alpha and ribavirin combination therapy.. Clin Endocrinol (Oxf).

[18] Dabrowska MM, Panasiuk A, Flisiak R (2010). Thyroid dysfunction in antiviral therapy of chronic hepatitis C.. Hepatogastroenterology.

[19] Friedrich-Rust M, Theobald J, Zeuzem S, Bojunga J (2009). Thyroid function and changes in ultrasound morphology during antiviral therapy with pegylated interferon and ribavirin in patients with chronic hepatitis C.. J Viral Hepat.

[20] Huang JF, Chuang WL, Dai CY, Chen SC, Lin ZY, Lee LP (2006). The role of thyroid autoantibodies in the development of thyroid dysfunction in Taiwanese chronic hepatitis C patients with interferon- alpha and ribavirin combination therapy.. J Viral Hepat.

[21] Jamil KM, Leedman PJ, Kontorinis N, Tarquinio L, Nazareth S, McInerney M (2009). Interferon-induced thyroid dysfunction in chronic hepatitis C.. J Gastroenterol Hepatol.

[22] Kee KM, Lee CM, Wang JH, Tung HD, Changchien CS, Lu SN (2006). Thyroid dysfunction in patients with chronic hepatitis C receiving a combined therapy of interferon and ribavirin: incidence, associated factors and prognosis.. J Gastroenterol Hepatol.

[23] Krupinska J, Wawrzynowicz-Syczewska M, Urbanowicz W, Poblocki J, Syrenicz A (2011). The influence of interferon alpha on the induction of autoimmune thyroiditis in patients treated for chronic viral hepatitis type C.. Endokrynol Pol.

[24] Masood N, Ghori R, Memon A, Memon S, Memon KI, Memon I (2008). Frequency of thyroid disorders during interferon and ribavirin therapy in chronic hepatitis C infection.. J Coll Physicians Surg Pak.

[25] Pavan MH, Pavin EJ, Goncales FL, Jr., Wittmann DE (2011). Virus C genotype predisposes to primary hypothyroidism during interferon- alpha treatment for chronic hepatitis C.. Braz J Infect Dis.

[26] Themistoklis V, Panagiotis A, Georgios N, Konstantinos S, Kaliopi P, Nikolaos G (2011). Thyroid dysfunction and long-term outcome during and after interferon-alpha therapy in patients with chronic hepatitis C.. Ann Acad Med Singapore.

[27] Tran HA, Reeves GE, Jones TL (2009). The natural history of interferonalpha2b- induced thyroiditis and its exclusivity in a cohort of patients with chronic hepatitis C infection.. Qjm.

[28] Vezali E, Elefsiniotis I, Mihas C, Konstantinou E, Saroglou G (2009). Thyroid dysfunction in patients with chronic hepatitis C: virus- or therapy-related?. J Gastroenterol Hepatol.

[29] Carella C, Mazziotti G, Amato G, Braverman LE, Roti E (2004). Clinical review 169: Interferon-alpha-related thyroid disease: pathophysiological, epidemiological, and clinical aspects.. J Clin Endocrinol Metab.

[30] Baudin E, Marcellin P, Pouteau M, Colas-Linhart N, Le Floch JP, Lemmonier C (1993). Reversibility of thyroid dysfunction induced by recombinant alpha interferon in chronic hepatitis C.. Clin Endocrinol (Oxf).

[31] Marazuela M, Garcia-Buey L, Gonzalez-Fernandez B, Garcia-Monzon C, Arranz A, Borque MJ (1996). Thyroid autoimmune disorders in patients with chronic hepatitis C before and during interferon- alpha therapy.. Clin Endocrinol (Oxf).

[32] Bini EJ, Mehandru S (2004). Incidence of thyroid dysfunction during interferon alfa-2b and ribavirin therapy in men with chronic hepatitis C: a prospective cohort study.. Arch Intern Med.

[33] Dalgard O, Bjoro K, Hellum K, Myrvang B, Bjoro T, Haug E (2002). Thyroid dysfunction during treatment of chronic hepatitis C with interferon alpha: no association with either interferon dosage or efficacy of therapy.. J Intern Med.

[34] Moncoucy X, Leymarie F, Delemer B, Levy S, Bernard-Chabert B, Bouche O (2005). Risk factors and long-term course of thyroid dysfunction during antiviral treatments in 221 patients with chronic hepatitis C.. Gastroenterol Clin Biol.

[35] Tran HA, Jones TL, Batey RG (2005). The spectrum of thyroid dysfunction in an Australian hepatitis C population treated with combination Interferon-alpha2beta and Ribavirin.. BMC Endocr Disord.

[36] Morisco F, Mazziotti G, Rotondi M, Tuccillo C, Iasevoli P, Del Buono A (2001). Interferon-related thyroid autoimmunity and long-term clinical outcome of chronic hepatitis C.. Dig Liver Dis.

[37] Tran HA, Attia JR, Jones TL, Batey RG (2007). Pegylated interferon-alpha- 2beta in combination with ribavirin does not aggravate thyroid dysfunction in comparison to regular interferon-alpha2beta in a hepatitis C population: meta-analysis.. J Gastroenterol Hepatol.

[38] Carella C, Mazziotti G, Morisco F, Manganella G, Rotondi M, Tuccillo C (2001). Long-term outcome of interferon-alpha-induced thyroid autoimmunity and prognostic influence of thyroid autoantibody pattern at the end of treatment.. J Clin Endocrinol Metab.

[39] Carella C, Mazziotti G, Morisco F, Rotondi M, Cioffi M, Tuccillo C (2002). The addition of ribavirin to interferon-alpha therapy in patients with hepatitis C virus-related chronic hepatitis does not modify the thyroid autoantibody pattern but increases the risk of developing hypothyroidism.. Eur J Endocrinol.

[40] Yan Z, Fan K, Wang Y, Fan Y, Tan Z, Deng G (2012). Changing Pattern of Clinical Epidemiology on Hepatitis C Virus Infection in Southwest China.. Hepat Mon.

[41] Gehring S, Kullmer U, Koeppelmann S, Gerner P, Wintermeyer P, Wirth S (2006). Prevalence of autoantibodies and the risk of autoimmune thyroid disease in children with chronic hepatitis C virus infection treated with interferon-alpha.. World J Gastroenterol.

[42] Kowala-Piaskowska A, Mozer-Lisewska I, Figlerowicz M, Sluzewski W (2007). Adverse effects during the treatment with pegylated interferon and ribavirin in children with chronic hepatitis C.. Pharmacoepidemiol Drug Saf.

[43] Kryczka W, Brojer E, Kowalska A, Zarebska-Michaluk D (2001). Thyroid gland dysfunctions during antiviral therapy of chronic hepatitis C.. Med Sci Monit.

[44] Nadeem A, Aslam M, Khan DA, Hussain T, Khan SA (2009). Effects of combined interferon alpha and ribavirin therapy on thyroid functions in patients with chronic hepatitis C.. J Coll Physicians Surg Pak.

[45] Parana R, Cruz M, Santos-Jesus R, Ferreira K, Codes L, Cruz T (2000). Thyroid disease in HCV carriers undergoing antiviral therapy with interferon plus ribavirin.. Braz J Infect Dis.

[46] Wirth S, Pieper-Boustani H, Lang T, Ballauff A, Kullmer U, Gerner P (2005). Peginterferon alfa-2b plus ribavirin treatment in children and adolescents with chronic hepatitis C.. Hepatology.

[47] Kabbaj N, Guedira MM, El Atmani H, El Alaoui M, Mohammadi M, Benabed K (2006). Thyroid disorders during interferon alpha therapy in 625 patients with chronic hepatitis C: a prospective cohort study.. Ann Endocrinol (Paris).

[48] Hsieh MC, Yu ML, Chuang WL, Shin SJ, Dai CY, Chen SC (2000). Virologic factors related to interferon-alpha-induced thyroid dysfunction in patients with chronic hepatitis C.. Eur J Endocrinol.

[49] Laurberg P, Pedersen KM, Hreidarsson A, Sigfusson N, Iversen E, Knudsen PR (1998). Iodine intake and the pattern of thyroid disorders: a comparative epidemiological study of thyroid abnormalities in the elderly in Iceland and in Jutland, Denmark.. J Clin Endocrinol Metab.

[50] Koh LK, Greenspan FS, Yeo PP (1997). Interferon-alpha induced thyroid dysfunction: three clinical presentations and a review of the literature.. Thyroid.

[51] Amir Z, Fatemeh E, Majid S (2009). Thyroid Dysfunction in Patients with Chronic Viral Hepatitis B and C during Alpha Interferon Therapy.. Hepat Mon.

[52] Preziati D, La Rosa L, Covini G, Marcelli R, Rescalli S, Persani L (1995). Autoimmunity and thyroid function in patients with chronic active hepatitis treated with recombinant interferon alpha-2a.. Eur J Endocrinol.

[53] Watanabe U, Hashimoto E, Hisamitsu T, Obata H, Hayashi N (1994). The risk factor for development of thyroid disease during interferon- alpha therapy for chronic hepatitis C.. Am J Gastroenterol.

[54] Ansar Ahmed S, Penhale WJ, Talal N (1985). Sex hormones, immune responses, and autoimmune diseases. Mechanisms of sex hormone action.. Am J Pathol.

[55] Gelu-Simeon M, Burlaud A, Young J, Pelletier G, Buffet C (2009). Evolution and predictive factors of thyroid disorder due to interferon alpha in the treatment of hepatitis C.. World J Gastroenterol.

[56] Antonelli A, Ferri C, Fallahi P (2009). Hepatitis C: thyroid dysfunction in patients with hepatitis C on IFN-alpha therapy.. Nat Rev Gastroenterol Hepatol.

